# Characterisation of HIV-1 Molecular Epidemiology in Nigeria: Origin, Diversity, Demography and Geographic Spread

**DOI:** 10.1038/s41598-020-59944-x

**Published:** 2020-02-26

**Authors:** Jamirah Nazziwa, Nuno Rodrigues Faria, Beth Chaplin, Holly Rawizza, Phyllis Kanki, Patrick Dakum, Alash’le Abimiku, Man Charurat, Nicaise Ndembi, Joakim Esbjörnsson

**Affiliations:** 1 Department of Translational Medicine, Lund University, Lund, Sweden; 2 Department of Zoology, University of Oxford, Oxford, United Kingdom; 3 Department of Immunology and Infectious Diseases, Harvard T.H. Chan School of Public Health, Boston, USA; 4 Institute of Human Virology Nigeria, Abuja, Nigeria; 5 Institute of Human Virology, University of Maryland School of Medicine, Baltimore, USA; 6 Nuffield Department Medicine, University of Oxford, Oxford, United Kingdom

**Keywords:** HIV infections, Viral infection

## Abstract

Nigeria has the highest number of AIDS-related deaths in the world. In this study, we characterised the HIV-1 molecular epidemiology by analysing 1442 HIV-1 *pol* sequences collected 1999–2014 from four geopolitical zones in Nigeria using state-of-the-art maximum-likelihood and Bayesian phylogenetic analyses. The main circulating forms were the circulating recombinant form (CRF) 02_AG (44% of the analysed sequences), CRF43_02G (16%), and subtype G (8%). Twenty-three percent of the sequences represented unique recombinant forms (URFs), whereof 37 (11%) could be grouped into seven potentially novel CRFs. Bayesian phylodynamic analysis suggested that five major Nigerian HIV-1 sub-epidemics were introduced in the 1960s and 1970s, close to the Nigerian Civil War. The analysis also indicated that the number of effective infections decreased in Nigeria after the introduction of free antiretroviral treatment in 2006. Finally, Bayesian phylogeographic analysis suggested gravity-like dynamics in which virus lineages first emerge and expand within large urban centers such as Abuja and Lagos, before migrating towards smaller rural areas. This study provides novel insight into the Nigerian HIV-1 epidemic and may have implications for future HIV-1 prevention strategies in Nigeria and other severely affected countries.

## Introduction

Thirty-eight years after the first AIDS case was described, HIV-1 remains a major public health problem that affects approximately 36.7 (30.8–42.9) million people globally^1,2^. Sub-Saharan Africa accounts for approximately 70% of all infections. In addition, HIV-1 represent one of the most fast-evolving pathogens known to science. This has led to the classification of HIV-1 into subtypes, sub-subtypes and circulating recombinant forms (CRFs). Of relevance, previous reports have suggested that the genetic composition of the infecting HIV-1 strain may influence transmission, disease progression, virus-host interactions, antiretroviral treatment responses, and vaccine development^3–12^. Moreover, it has been suggested that targeted HIV-1 prevention may be a cost-effective way to decrease the number of new HIV-1 infections – not least in low-and-middle-income countries^13^. Planning of such HIV-1 intervention programs will require detailed information about both the characteristics and sources of new infections.

Nigeria is the most populous country in Sub-Saharan Africa, has the second highest number of HIV-1 infected persons in the world, and the highest number of annual AIDS-related deaths^14,15^. HIV-1 serological surveys in Nigeria were initiated in 1991, at which time the adult prevalence was 1.8% (760000 people)^16^. This figure increased to 5.8% by 2001 (2.6 million), before declining to 2.8% in 2017 (3.1 million)^2,16^. Previous reports have identified subtype G and the CRF02_AG as the most common HIV-1 variants in Nigeria^17–20^. In addition, the CRF43_02G was recently shown to be highly prevalent in the capital Abuja^21^. However, the estimates of each strain’s contribution to the Nigerian HIV-1 epidemic have varied considerably, with frequency estimates ranging from 22–50% for subtype G, and 19–60% for the CRF02_AG^18–25^. These variations could have several reasons, such as differences between geographic areas, transmission groups, or employed subtyping tools^26,27^. Hence, a clearer picture of the HIV-1 subtype/CRF distributions and spread in Nigeria is needed. The objective of the current study was therefore to perform the first nationwide analysis of the molecular epidemiology of HIV-1 in Nigeria.

## Methods

### Sequence dataset

We analyzed 366 previously unpublished HIV-1 *pol* sequences (positions in HXB2 K03455: 2253–3364) collected in Abuja, Nigeria between 2006–2011, together with all Nigerian *pol* sequences from the corresponding genetic region available in the Los Alamos HIV-1 sequence database (N = 1076, http://www.hiv.lanl.gov/, Table 1). All sequences without date and location of sampling were excluded. However, contact with all relevant research centers was made in order to obtain such missing information.

**Table 1:** Proportion of subtype/CRF in the Nigerian dataset of previously published and new sequences collected in the period 1999–2013.

Subtype/CRF^a^	N^c^	%	Geography^d^																	
			Southwest							North Central					North East & North West					
			LAG	IBA	OND	OSO	OYO	ENU	Total	ABV	JOS	MIN	NC	Total	KAD	DAM	MAI	KAN	YOL	Total
A1	24	1.7	9	2	0	0	0	0	**11**	7	5	0	0	**12**	0	0	1	0	0	**1**
B	4	0.3	1	1	0	0	0	0	**2**	2	0	0	0	**2**	0	0	0	0	0	**0**
C	14	1.0	2	1	0	0	2	0	**5**	6	2	0	1	**9**	0	0	0	0	0	**0**
CRF02_AG	636	44.1	176	27	0	1	18	0	**222**	284	94	0	10	**388**	13	0	11	2	0	**26**
CRF06_cpx	64	4.4	18	2	0	0	0	0	**20**	36	6	0	1	**43**	0	0	1	0	0	**1**
CRF09_cpx	1	0.1	0	1	0	0	0	0	**1**	0	0	0	0	**0**	0	0	0	0	0	**0**
CRF11_cpx	4	0.3	1	0	0	0	0	0	**1**	2	1	0	0	**3**	0	0	0	0	0	**0**
CRF18_cpx	1	0.1	1	0	0	0	0	0	**1**	0	0	0	0	**0**	0	0	0	0	0	**0**
CRF19_cpx	1	0.1	0	0	0	0	0	0	**0**	0	1	0	0	**1**	0	0	0	0	0	**0**
CRF43_02G	236	16.4	33	11	1	0	0	0	**45**	138	34	0	0	**172**	8	0	8	3	0	**19**
CRF49_cpx	1	0.1	1	0	0	0	0	0	**1**	0	0	0	0	**0**	0	0	0	0	0	**0**
D	9	0.6	2	0	0	0	0	0	**2**	4	2	0	0	**6**	0	0	1	0	0	**1**
G	119	8.3	24	6	0	0	0	0	**30**	55	25	1	0	**81**	4	1	3	0	0	**8**
URF^b^	328	22.7	78	13	0	0	2	1	**94**	163	46	0	3	**212**	9	0	11	0	2	**22**
Total	**1442**	100	346	64	1	1	22	1	**435**	697	216	1	15	**929**	34	1	35	5	2	**78**

^a^CRF, Circulating recombinant forms; ^b^URF, Unique recombinant forms; ^c^N, number of sequences; ^d^Abbreviations for the different cities: LAG, Lagos; IBA, Ibadan; OND, Ondo; OSO, Osogbo; OYO, Oyo; ENU, Enugu; ABV, Abuja; JOS, Jos; MIN, Minna; NC, Other North central cities; KAD, Kaduna; DAM, Damaturu; MAI, Maiduguri; KAN, Kano; YOL, Yola.

### Subtype determination

The Nigerian *pol* sequences were aligned with all M group lineages (A-K + Recombinants) of the LANL 2010 Reference Sequence Dataset (http://www.hiv.lanl.gov/) using the Clustal algorithm, followed by manual editing in MEGA6^28,29^. The HIV-1 subtype/CRF classification was determined by manual assignment with maximum-likelihood (ML) phylogenetic analysis in Garli v0.98^30^ using the General Time Reversible (GTR) substitution model. Branch support was determined in PhyML 3.0 using the approximate likelihood ratio test with the Shimodaira-Hasegawa-like procedure (aLRT-SH)^31^. Branches with aLRT-SH support >0.90 were considered statistically supported^32^.

### Recombination analysis

Putative unique recombinant forms (URFs) and sequences that were difficult to subtype were analysed by Bootscan in Simplot^33^. Briefly, *pol* sequences were aligned with the LANL 2010 Reference sequences for subtypes for G, CRF4302G and CRF02AG as putative parental sequences. Recombination breakpoints were identified using a sliding window size of 300 bp and step size of 50 bp. URFs consisting of non-subtype G related forms were excluded from the Simplot analysis.

In order to define the structure and distribution of the breakpoints across the alignment, we plotted a line graph of the relative frequency of the breakpoints. The K-means univariate-clustering algorithm as implemented in the ‘Ckmeans.1d.dp’ R package was used to define hotspots for recombination^34^. The gap statistic method implemented in the ‘factoextra’ R-package was employed to estimate the groups based on similarity in breakpoint positions obtained from the Simplot analysis^35^. The recombination hotspot positions were then used to identify groups of three or more URFs with one or more similar breakpoint positions. Finally, to determine potential new CRFs, we performed a ML phylogenetic analysis using Garli (GTR nucleotide substitution model) to assess the epidemiological relatedness among the sequences^36^.

### Cluster analysis

A previously described BLAST approach was used to determine non-Nigerian subtype/CRF-specific reference sequences^32,37^. Subtype/CRF-specific reference sequences and Nigerian sequences were aligned and ML phylogenies determined as described previously^32^. Nigerian transmission clusters were defined as clusters with aLRT-SH support ≥0.90 and composed of ≥80% Nigerian sequences^32,38^. Clusters of two sequences were defined as dyads, 3–14 sequences as networks, and >14 sequences as large clusters^39^.

### Dated phylogeographic analysis

The temporal signal in each dataset were assessed by estimating the regression between divergence and sampling dates in TempEst^40^. For the main Nigerian transmission clusters with little or no temporal structure, we used substitution rate priors obtained by preliminary analysis of 150 randomly selected sequences sampled worldwide for each subtype/CRF. The evolutionary rates were estimated in BEAST v1.8.4^41^ using the SRD06 model^42^, a relaxed uncorrelated lognormal clock model^43,44^, and the Bayesian Skygrid coalescent tree prior^45^. For each transmission cluster, Markov chain Monte Carlo (MCMC) chains were run for 300 million steps, subsampling parameters and trees every 30000th step. BEAGLE library v.2 was used to improve computational time of likelihood calculations^46^. Convergence was assessed using Tracer v.1.6. All parameters achieved convergence as determined by effective sample sizes (ESS) ≥100^47^.

We used a Bayesian discrete phylogeographic approach with a MCMC length of 300 million steps in BEAST v1.8.4, sampling every 30000th step, to reconstruct the spatial dynamics of HIV-1 for the identified large clusters^41,48^. Effective population sizes through time was inferred using the Bayesian Skygrid coalescent model^49^. Growth rates were estimated using the parametric exponential growth rate coalescent model^50,51^.

Symmetric and asymmetric continuous time Markov chain models were used to model the location exchange process^52^. The most parsimonious description of the location exchange rates was inferred using the Bayesian stochastic search variable selection (BSSVS) procedure^52^. A robust counting approach as implemented in BEAST was used to estimate the forward and reverse viral movement events between locations along the branches of time dated phylogenetic trees^53^. Well-supported movements were summarized using SPREAD v1.0.7 based on a Bayes factors ≥3^54–56^. The percentage of viral movements between locations was summarized using R^57^. All files and scripts are available upon request.

### Statistics

Linear by linear association test (LBL) was used to analyze trends over time, using IBM SPSS V22.0 Armonk, NY: IBM Corporation.

### Ethics

All research was performed in accordance with relevant guidelines/regulations, and informed consent was obtained from all participants if appropriate. Specifically, the study utilized secondary data analysis from laboratory database of the National HIV program, which has approval from the Nigerian National Health Research Ethics Committee (NHREC Approval #NHREC/01/01/2007-14/08/2017).

### Accession numbers for previously unpublished sequences used in this study

The DNA sequences of HIV-1 *pol* PR-RT regions determined as part of this study were submitted to GenBank under the following accession numbers: DQ990400 to DQ990455.

## Results

### CRF02_AG, CRF43_02G and subtype G were the major circulating strains in Nigeria

We analyzed 1442 HIV-1 *pol* sequences collected from four geopolitical zones in Nigeria between 1999 and 2013 (Table 1). The phylogenetic analysis showed that the CRF02_AG was the most common strain (44% of the analyzed sequences), followed by CRF43_02G (16%), subtype G (8%) and CRF06_cpx (4%). Three-hundred-and-twenty-eighty of the sequences (23%) were unique recombinant forms (URFs), whereas the remaining sequences were minor variants (each variant accounting for <2%).

**Figure 1:**
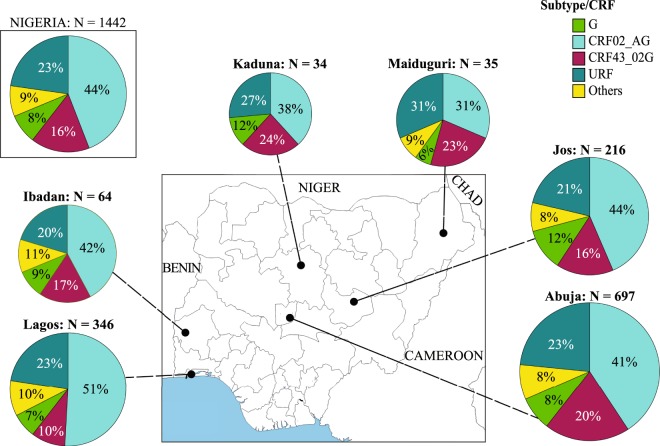
Prevalence of subtypes/CRFs among HIV-1 infected individuals collected from different locations in Nigeria. HIV-1 diversity in different zones/regions in Nigeria. The locations of the sampled regions as indicated on the map are connected to their respective diversity pie charts by a dot-headed dashed black line.

Most sequences were from Abuja (697 sequences, 48%), followed by Lagos (346 sequences, 23%) and Jos (216 sequences, 15%). The distribution of different subtypes/CRFs varied within these regions/states with fewer CRF02_AG infections following a North East direction from Lagos (Fig. 1). Analysis of trends over time showed an overall decrease in the proportion of CRF02_AG infections in Nigeria (from 55% in 2006 to 38% in 2013, p = 0.015, LBL, Supplementary Fig. S1). Moreover, the analysis also showed an increase in the proportion of unique recombinant forms (URFs) from 16% to 32%, 2005–2013 (p = 0.015, LBL). The time trend analysis for other CRFs and or subtypes was non-significant.

### Four potential recombination hotspots in the *pol* region

Of the 328 identified URFs, 209 constituted variations of the three dominating circulating strains in Nigeria (CRF02_AG, CRF43_02G, and subtype G; Supplementary Fig. 4). The remaining 119 URFs consisted of different combinations of various HIV-1 strains less common in Nigeria. We therefore performed a detailed recombination analysis on the 209 URFs consisting of CRF02_AG, CRF43_02G, and subtype G. One-hundred of the 209 URF sequences (48%) were from Abuja; 45 from Lagos (22%); 35 from Jos (17%); 10 from Maiduguri (5%); 9 from Kaduna (4%); seven from Ibadan (3%); and two from Adamawa (1%); and one from Enugu (<1%). These sequences were initially selected from the maximum likelihood phylogenetic trees if they branched off close to the root between two subtypes and had long branches. There were 655 breakpoint positions recorded among the 209 sequences, with some sequences having more than one breakpoint. These positions were plotted as a frequency plot of breakpoints along the alignment to identify hotspots for recombination (i.e. positions in the alignment were recombination breakpoints occur more frequently than the other positions, Supplementary Fig. S2). Alignment positions around 285–315 (HXB2 K03455 positions: 2538–2568), 503–534 (2756–2787), 720–775 (2973–3028) and 923–956 (3176–3209) were identified as potential recombination hotspots. To define these positions more precisely, we used the inter-quartile range (IQR) of the optimal univariate K-median clustering algorithm with the number of clusters determined by the gap-statistic method (Supplementary Fig. S3)^58^: Recombination hotspot I: 294–312 (HXB2 K03455 positions: 2547–2565); II: 503–533 (2756–2786); III: 729–805 (2982–3058); and IV: 931–957 (3184–3210) (Supplementary Table S1). Of the 209 sequences, 139 (67%) had a recombination breakpoint in the hotspot I region; 104 (50%) in hotspot II region; 58 (28%) in hotspot III region; and 59 (28%) in hotspot IV region. The hotspot positions were unique independent recombination events from the original breakpoint positions as previously identified for the parental sequences of CRF43_02G (HXB2 K03455 positions: 1266, 3325, and 6097) and CRF02_AG (HXB2 K03455 positions: 2391, 3275, and 4175). We identified 37 URFs that could be divided into seven groups sharing breakpoint positions (each group consisting of 3–10 sequences, respectively, and thereby fulfilling the requirements as potentially novel CRFs; Supplementary Table S2). The majority of these sequences had been collected in Abuja (23/37, 62%).

### Cluster analysis

To determine transmission clusters of the major circulating strains within Nigeria, we analyzed the three dominating forms: subtype G, CRF02_AG, and CRF43_02G. Analysis of 206 subtype G sequences (119 Nigerian and 87 non-Nigerian) showed four dyads, one network, and one large Nigerian subtype G cluster (consisting of 81 Nigerian and 11 non-Nigerian sequences, Table 2 and Supplementary Fig. S5). The 1161 CRF02_AG sequences (636 Nigerian and 555 non-Nigerian) formed 12 dyads, 12 networks and six clusters (Supplementary Fig. S6). Finally, analysis of the 295 CRF43_02G sequences (236 Nigerian and 59 non-Nigerian) showed that all the Nigerian sequences clustered monophyletically (SH-aLRT = 0.99, Supplementary Fig. 7), suggesting that CRF43_02G predominately circulates in Nigeria.

**Table 2:** Number of clusters in the different subtype/CRF groups.

Subtype/CRF	Dyads^a^	Networks^b^	Large clusters^c^	Total
G	8 (4)	8 (2)	94 (1)	104
CRF02_AG	24 (12)	76 (12)	336 (6)	439
CRF43_02G	0 (0)	0 (0)	295 (1)	295
**Total**	**32**	**84**	**725**	**841**

^a^Dyads: Clusters of 2 sequences.

^b^Networks: Clusters of 3–14 sequences.

^c^Large clusters: Clusters with more than 14 sequences.

The number of clusters observed for the different groups are indicated in brackets.

### Dates of origin and evolutionary rates of the main Nigerian transmission clusters

To further dissect the Nigerian HIV-1 epidemic, we performed a detailed analysis of the five largest Nigerian clusters (one subtype G, three CRF02_AG, and one CRF43_02G). The time to most recent common ancestor (tMRCA) of the Nigerian subtype G cluster was 1975 (95%HPD: 1969–1982). The corresponding estimates for the CRF02_AG clusters were 1963 (95%HPD: 1948–1974), 1970 (95%HPD: 1960–1980) and 1960 (95%HPD: 1947–1974) for cluster 1, 2 and 3, respectively; and 1971 (95%HPD: 1952–1983) for the CRF43_02G cluster (Supplementary Fig. 8). The HIV-1 evolutionary rate for the Nigerian subtype G cluster was 2.1 × 10^−3^ substitutions/site/year (s/s/y, 95%HPD: 1.67–2.53 × 10^−3^ s/s/y). The rates for the CRF02_AG clusters 1, 2 and 3 was slightly lower: 1.42 × 10^−3^ s/s/y (95% HPD: 1.07–1.85 × 10^−3^ s/s/y) for cluster 1; 1.34 × 10^−3^ s/s/y (95%HPD: 1.02–1.72 × 10^−3^ s/s/y) for cluster 2; and 1.22 × 10^−3^ s/s/y (95%HPD: 0.91–1.57 × 10^−3^ s/s/y) for cluster 3. Finally, the rate for the CRF43_02G cluster was estimated to 2.72 × 10^−3^ s/s/y (95%HPD: 1.59–3.73 × 10^−3^ s/s/y) (Supplementary Fig. 8).

To assess the impact of sampling bias in our phylogeographic inference, we conducted a control analysis of the effects of potential over-representations of samples from particular regions in Nigeria (Table 1). Sequences were randomly selected based on population growth over time and HIV-1 prevalence in the different geographic regions. Information on the Nigerian population growth over time was obtained from the National Population Commission of Nigeria/National Bureau of Statistics for the population census, and information on HIV-1 prevalence was obtained from National Agency for the Control of AIDS^16^. In these analyses, the median tMRCA of the Nigerian subtype G cluster was estimated to 1987 (95%HPD: 1982–1992); and the CRF02_AG clusters to 1974 (95%HPD: 1960–1983), 1972 (95%HPD: 1973–1981), 1961 (95%HPD: 1952–1979) for cluster 1, 2 and 3, respectively. Despite numerous attempts, the CRF43_02G control analysis did not reach sufficient temporal signal to converge.

### Disentangling the demographic history of the main Nigerian transmission clusters

**Figure 2:**
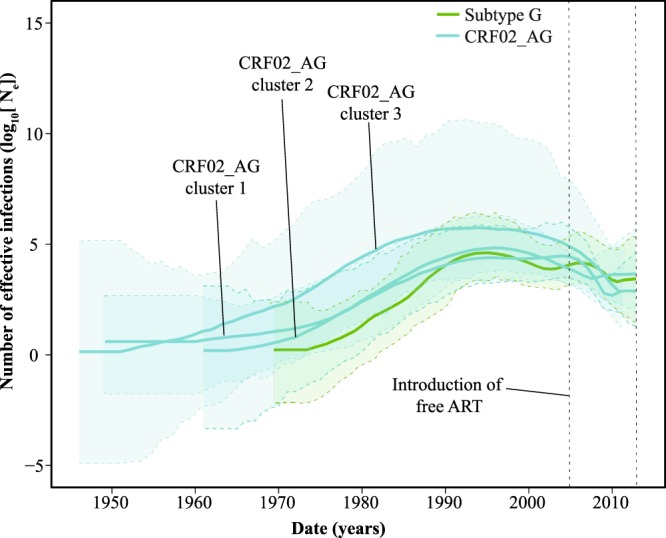
Skygrid plots for the different clusters. Phylodynamic analyses of HIV-1 *pol* gene for subtype G and CRF02_AG isolated in Nigeria. Bayesian Skygrid plots representing the changes in the effective population size of the virus (*N*_*e*_) over time. The solid lines represent the estimated median log effective population size and the colour-coded shaded areas represent the 95%HPD intervals for the different clusters circulating in Nigeria. The gray-shaded area starts at the period when ART was introduced in Nigeria.

**Figure 3:**
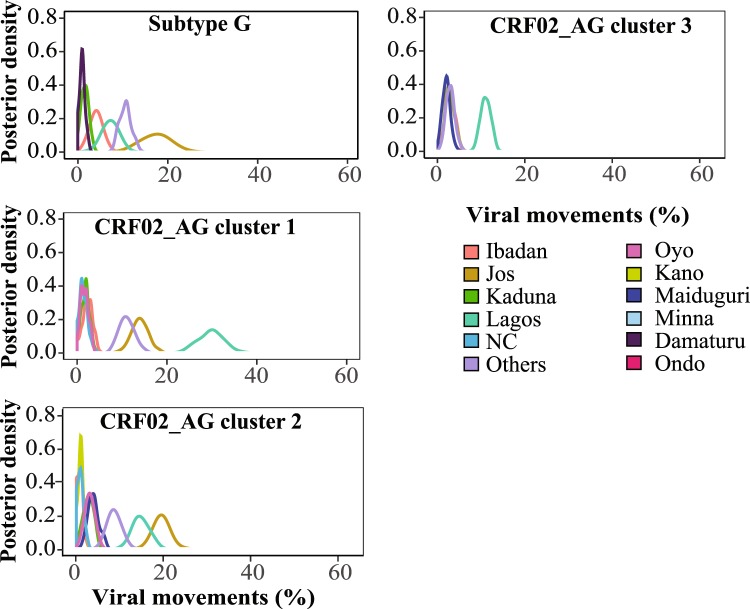
Estimated percentages of viral migration events from Abuja to each location in Nigeria. The density plot for the viral movements from the Abuja (most probable root location) to Lagos, Jos, Kaduna and other towns. Lagos had the highest percentage of viral movements from Abuja for the CRF02_AG cluster 1, and CRF02_AG cluster 3; whereas Jos had the highest percentage of viral movements from Abuja for CRF02_AG cluster 2 and subtype G.

The analysis of the Nigerian subtype G epidemic showed that the number of effective infections, a proxy of HIV incidence, underwent a fast exponential growth between the 1970s and the mid-1990s, followed by a marginal decrease with minor fluctuations from the mid-1990s and onwards (Fig. 2)^59^. Using the exponential growth model, the median growth rate was 0.3 per year (95%HPD: 0.18–0.42). The three clusters representing the CRF02_AG epidemic displayed a similar pattern with a slow increase in growth rate from the 1980s to 2000s. The median CRF02_AG growth rates were estimated to 0.22 per year (95%HPD: 0.13–0.32) for cluster 1; 0.18 per year (95%HPD: 0.10–0.26) for cluster 2; and 0.24 (95%HPD: 0.11–0.38) for cluster 3 (Fig. 2). Finally, the CRF43_02G cluster had a median growth rate of 0.39 per year (95%HPD: 0.23–0.55). The CRF43_02G cluster had low temporal signal that could not inform the complex non-parametric coalescent model and thus only an exponential model was used to capture the demographic dynamics (Fig. 3).

### Phylogeographic dispersal of HIV-1 in the main Nigerian subepidemics

Next, we sought to investigate the spatiotemporal spread of HIV-1 in Nigeria using both asymmetric and symmetric discrete phylogeographic diffusion models. From the asymmetric analysis, various sample locations (cities) had well supported exchange rates that dominated the diffusion process of the different subepidemics. For subtype G, significant transition between Lagos and Ibadan (Bayes Factor [BF] = 241), Damaturu (BF = 109), Minna (BF = 174), and Jos (BF = 98) were identified. Frequently invoked rates were also identified between Abuja and Lagos (BF = 81), Kaduna (BF = 220), Ibadan (BF = 223), Damaturu (BF = 293) and Minna (BF = 138). Supported rates from Kaduna, Jos and Ibadan to the other locations within Nigeria were also found. Finally, the analysis also indicated significant international linkages to and from Lagos (BF = 74), Abuja (BF = 80), Kaduna (BF = 21), Jos (BF = 501), Damaturu (BF = 80), and Ibadan (BF = 207). The symmetric models gave similar results as the asymmetric model.

For the CRF02_AG cluster 1, Abuja was connected to Ibadan (BF = 105), Kaduna (BF = 687), and Jos (BF = 116); Lagos was connected to Ibadan (BF = 512), Kaduna (BF = 134) and Oyo (BF = 139); Kaduna was connected to Jos (BF = 207), Ibadan (BF = 371) and Oyo (BF > 1000). International linkage was also found for Lagos (BF = 639), Abuja (BF = 184), Ibadan (BF = 462) and Oyo (BF = 275). For CRF02_AG cluster 2, Lagos was connected to Jos (BF = 395), Maiduguri (BF = 231), Ibadan (BF > 1000) and Kaduna (BF = 383); Abuja to Kaduna (BF = 155), Ibadan (BF = 110), Kano (BF = 543) and Oyo (BF = 58); and Ibadan to Kaduna, Jos, Oyo, Kano, and Maiduguri. For the CRF02_AG cluster 3, Lagos was connected to Abuja (BF = 118), Jos (BF = 87) and Maiduguri (BF = 56). Abuja was connected to Ibadan (BF = 45), Jos (BF = 44) and Maiduguri (BF = 29).

As described above, the CRF43_02G cluster had low temporal signal and could not inform the complex phylogeographic analysis. Finally, based on posterior probability support, the location of the spatial origin of subtype G, and CRF02_AG clusters was Abuja (posterior probability ≥ 0.98).

### Rates of viral lineage migration

The rates of viral lineage migration within Nigeria were estimated using a robust counting approach. For subtype G, 42% (95%HPD: 35–48%) of all virus migrations originated in Abuja; of these 17% (95%HPD: 12–22%) were directed to Jos, 10% (95%HPD: 8–13%) to outside Nigeria, and 7% (95%HPD: 4–10%) to Lagos. Similar results were found for the CRF02_AG clusters, except for slightly higher migration rates from Abuja to Lagos for cluster 1 (30%, 95%HPD: 25–34%, Fig. 3). Overall, the CRF02_AG migration rates originating in Abuja was 61%, 52% and 21% for cluster 1, 2 and 3, respectively.

## Discussion

In this study, we aimed to disentangle the history and spread of HIV-1 subtypes/CRFs in Nigeria using state-of-the-art phylogenetics to a large set of HIV-1 sequences collected in the five most populated Nigerian geopolitical zones. To the best of our knowledge, only one previous nationwide molecular epidemiology study from Nigeria have been presented^60^. In this study, 55 HIV-1 *gp41* sequences (approximately 400 bp) collected throughout Nigeria during 1999 were analysed by neighbour-joining phylogenetics. However, the analysis was unable to distinguish between subtype A and CRF02_AG sequences – likely due to insufficient phylogenetic information in this relatively short gene fragment, as suggested by the authors. In-depth phylogenetic and phylodynamic analysis was therefore not possible in this study. In comparison, we analysed 1442 HIV-1 *pol* sequences (approximately 1000 bp long) collected throughout Nigeria between 1999 and 2013 using maximum-likelihood and Bayesian phylogenetics. Importantly, the HIV-1 *pol* gene (which is the most targeted genetic region for HIV-1 sequencing and by far most commonly analysed region in studies of HIV-1 molecular epidemiology) holds sufficient intrinsic genetic variability to permit the reconstruction of transmission histories by phylogenetic approaches^26,32,61^. Hence, the larger size of our dataset, the relatively long time-window of sampling, and the use of state-of-the-art phylodynamic methods enabled us to perform the first in-depth nationwide HIV-1 phylodynamic study in Nigeria. In line with other previous studies from different geographic regions in Nigeria, we found that CRF02_AG, CRF43_02G and subtype G were the most prevalent strains (previous estimates ranging from 19–60% for CRF02_AG and 22–50% for subtype G^18–25,62^). The prevalence of CRF43_02G has only been reported in one previous study from Abuja (estimated to 19%)^21^. Non-representative sampling or local fluctuations between geographic regions in Nigeria could explain the large variations and discrepancies between studies. It could also be explained, in part, by the use of different subtyping tools between studies (which can differ in accuracy in assigning the correct subtype/CRF based on partial genome sequences)^27^.

We found one large well-supported monophyletic cluster for both subtype G and CRF43_02G, respectively. Each of these clusters harbored the majority of Nigerian sequences, suggesting single strain introductions that grew out to dominate the Nigerian HIV-1 epidemic. Previous studies have used different nomenclatures of subtype G. A comparison of accession numbers showed that our subtype G cluster is partly consistent with the subtype G’ cluster identified by Chaplin *et al*.^22^. Further dissection of the subtype G’ cluster showed that approximately half of the sequences belonged to subtype G, whereas the other half clustered with the CRF43_02G sequences in our analysis. In a more recent study, Delatorre *et al*. suggested a nomenclature based on geographic dissemination^63^. The clustering pattern of the West African strain defined as subtype G_WA-II_ was largely overlapping with the Nigerian subtype G cluster in our analysis^63^. We estimated the date of origin for the subtype G cluster to 1975 (95%HPD: 1969–1982), which is consistent with the origin of the subtype G_WA-II_ cluster that was estimated to 1979 [95%HPD: 1973–1984])^63^. In addition, the estimated date of origin also fits with the fact that the first AIDS cases were identified in Nigeria in 1986, approximately one decade after the introduction of this virus strain^16^. The CRF43_02G was first described and isolated in Saudi Arabia in 2008^64^. Interestingly, the G_WA-I_ cluster determined by Delatorre *et al*. consisted of 140/168 (83%) sequences from Nigeria^63^. In our analysis, more than half of the G_WA-I_ sequences clustered with the CRF43_02G strain. Spatiotemporal analysis of the G_WA-I_ cluster indicated that this strain emerged in Nigeria in the mid-1970’s^63^. This is consistent with our estimate of 1971 (95%HPD: 1952–1983) for the CRF43_02G cluster. Moreover, the majority of CRF43_02G sequences in Genbank are of Nigerian origin (www.hiv.lanl.gov), and both the putative parental strains of CRF43_02G in Nigeria (CRF02_AG and subtype G) are highly prevalent. Altogether, this suggests that CRF43_02G originated in central West Africa, in or close to Nigeria.

In contrast to subtype G and CRF43_02G, where only one of the respective introductions resulted in major subepidemics, three large Nigerian subepidemics were found for CRF02_AG. The underlying mechanisms for this strain-specific difference are unknown. However, the CRF02_AG strain is the most prevalent strain in West Africa, and a previous study of the West Central African CRF02_AG epidemic estimated that this strain originated in the Democratic Republic of the Congo (DRC) in 1973 (95%HPD: 1972–1975)^65^. This is in line with a previous study by Abecasis *et al*. that estimated the CRF02_AG date of origin to 1976 (95%HPD: 1971–1981)^66^. However, a more detailed analysis of the CRF02_AG strain by Mir *et al*. indicated that seven subvariants of CRF02_AG are circulating (as determined by a combination of phylogenetics and geographic dissemination)^67^. The seven subvariants of CRF02_AG were estimated to have originated between 1967 and 2003 (combined 95%HPD: 1961–2005). More specifically, the analysis indicated that the oldest strain was the West African CRF02_AG strain (introduced 1967 [95%HPD: 1961–1974]). The three CRF02_AG clusters identified in the current study were estimated to have originated between 1963 and 1970 (combined 95%HPD: 1948–1980). Although the 95%HPD intervals are overlapping with the estimates suggesting the origin to be the DRC, they are on the lower side. Moreover, the CRF02_AG strain was first isolated in Nigeria in 1996^68^. Due to the somewhat conflicting data presented above, further analysis is needed to provide solid evidence of the geographical origin of the CRF02_AG strain.

Bayesian demographic analysis indicated an increase in the number of effective infections 1970–1995 in all the analysed clusters. Interestingly, a rapid population growth occurred in Nigeria during the same period (from 56 to 108 million people, www.worldometers.info/world-population/nigeria-population). This increase was followed by a decline in number of effective infections that coincided with the introduction of free ART in Nigeria in 2006, which resulted in a decrease in HIV-1 prevalence from 6% to 3% in Nigeria the following years^2,16^. Moreover, our analysis suggested that urban areas like Abuja and Lagos were the major hubs of HIV-1 transmission in Nigeria. This is in line with previous reports from West Africa^69^. However, it should be noted that the majority of the sequences were collected in Abuja, and potential transmissions from other cities may therefore not have been captured in our analysis.

The recombination analysis indicated several URFs and potentially novel CRFs. Recombination does not occur randomly on the HIV-1 genome as its frequency varies along its length with several so-called hotspots for recombination^70,71^. One main hotspot for recombination (found in 105 sequences) was close to the *pol PR-RT* border (positions 2547–2565 in the HIV-1 HXB2 reference genome, K03455). This hotspot has previously been reported in a study on HIV-1 subtype B^72^. The hotspot positions II and IV have also been reported previously^73^. To date, eighteen distinct subtype G-related CRFs have been described (www.hiv.lanl.gov). We identified seven groups of URFs with similar recombination breakpoint patterns. These could represent novel CRFs or second-generation recombinants circulating in Nigeria. However, full-length genome sequencing is needed to confirm whether these groups represent similar URFs or previously unknown subtype G-related CRFs.

In summary, this is the first in-depth HIV-1 phylodynamic study based on a nationwide set of HIV-1 sequences from Nigeria. We found a high number of URFs and potential new CRFs, and our analyses suggested that HIV-1 first emerged and expanded within large urban centers before migrating to smaller rural areas. The number of effective infections declined in the early 2000s, coinciding with the introduction of free ART in Nigeria. This study increases our understanding of the Nigerian HIV-1 epidemic and may inform HIV-1 intervention strategies to reduce the spread of HIV-1 in Nigeria.

## Supplementary information

Supplementary Information.
