# Supplementary material for: Characterisation of HIV-1 Molecular Epidemiology in Nigeria: Origin, Diversity, Demography and Geographic Spread
Source: Sci Rep. 2020 Feb 26;10:3468. doi: 10.1038/s41598-020-59944-x (PMC7044301; doi:10.1038/s41598-020-59944-x)

Characterisation of HIV-1 Molecular Epidemiology in Nigeria: Origin, Diversity, Demography and Geographic Spread

Jamirah NAZZIWA[a], Nuno Rodrigues FARIA[b], Beth CHAPLIN[c], Holly RAWIZZA[c], Phyllis KANKI[c] Patrick DAKUM[d,e], Alash'le ABIMIKU[d,e], Man CHARURAT[e], Nicaise NDEMBI[d] and Joakim ESBJÖRNSSON[a,f#]

[a]Department of Translational Medicine, Lund University, Lund, Sweden

[b]Department of Zoology, University of Oxford, Oxford, United Kingdom

[c]Department of Immunology and Infectious Diseases, Harvard T.H. Chan School of Public Health, Boston, USA

[d]Institute of Human Virology Nigeria, Abuja, Nigeria

[e]Institute of Human Virology, University of Maryland School of Medicine, Baltimore, USA

[f]Nuffield Department Medicine, University of Oxford, Oxford, United Kingdom

#Address correspondence to:

Joakim Esbjörnsson

Systems Virology, Department of Translational Medicine, Lund University

BMC B13

221 84 Lund, Sweden

E-mail: joakim.esbjornsson@med.lu.se

SUPPLEMENTAL DIGITAL CONTENT

<u>Files in this Data Supplement:</u>

Supplementary Table S1. Summary statistics for the four hotspot regions in the HIV-1 *pol* alignment using the univariate K-means clustering algorithm.

Legends for supplementary figures

Supplementary Figure S1. Dynamics of subtype/CRF proportions, ART coverage and overall country HIV-1 prevalence over time.

Supplementary Figure S2. Potential recombination breakpoint hotspots detected in the pol alignment.

Supplementary Figure S3. Optimal clusters determined by the gap statistic method.

Supplementary Figure S4. Groups of sequences with similar recombination breakpoint patterns that could be potential new CRFs.

Supplementary Figure S5. Maximum likelihood phylogenetic tree for Subtype G sequences.

Supplementary Figure S6. Maximum likelihood phylogenetic tree for CRF02_AG sequences.

Supplementary Figure S7. Maximum likelihood phylogenetic tree for CRF43_02G sequences.

Supplementary Figure S8. Demographic history for the different Nigerian clusters.

Supplementary Table S1. Summary of the median, mean and interquartile ranges for the four hotspot regions in the HIV-1 *pol* alignment using the univariate K-means clustering algorithm.

	I	***II***	***III***	***IV***
**Minimum**	147.00	415.00	656.00	864.00
**1st Quartile**	294.00	503.00	729.00	931.00
**Median**	305.00	522.00	768.00	948.00
**Mean**	306.38	519.96	760.44	941.24
**3rd Quartile**	312.00	533.00	805.00	957.25
**Maximum**	398.00	633.00	858.00	980.00
**Number**	221	203	115	116

Supplementary Table S2. The putative CRFs identified in this study and their repsective genetic composition and geographical distribution.

	Number	Subtype/CRF in regions between hotspots (HXB2 positions, K03455):					City of collection (number and proportion of sequences)					
		2253-2558	*2559-2775*	*2776-3021*	*3022-3201*	*3202-3364*	Abuja	Adamwa	Ibadan	Jos	Kaduna	Lagos
**Putative CRF type 1**	10	CRF43_02G	G	CRF43_02G	CRF43_02G	CRF43_02G	6 (60%)			1 (60%)	3 (60%)	
**Putative CRF type 2**	3	CRF43_02G	CRF02_AG	CRF43_02G	CRF43_02G	CRF43_02G	1 (33%)		1 (33%)		1 (33%)	
**Putative CRF type 3**	5	CRF43_02G	CRF02_AG	CRF02_AG	CRF43_02G	CRF02_AG	4 (80%)					1 (20%)
**Putative CRF type 4**	7	CRF43_02G	CRF02_AG	CRF02_AG	CRF43_02G	G	4 (57%)	1 (14%)	1 (14%)			1 (14%)
**Putative CRF type 5**	4	CRF43_02G	G	CRF43_02G	CRF02_AG	CRF02_AG	3 (75%)		1 (25%)			
**Putative CRF type 6**	4	CRF43_02G	G	G	CRF43_02G	G	3 (75%)					1 (25%)
**Putative CRF type 7**	4	CRF43_02G	G-like	G-like	CRF43_02G	CRF02_AG	1 (25%)					3 (75%)

Legends for supplementary figures

Supplementary Figure S1. Dynamics of subtype/CRF proportions, ART coverage and overall country HIV-1 prevalence over time.

The subtype/CRF proportion displayed as overall percentage of sequences collected from Nigeria per year. Few sequences (<20 per year) were collected from 1999-2004 and their proportions had no effect on the LBL association tests. No sequences were collected in 2012. The orange bars represent the proportion of HIV-1 infected individuals that were receiving ART in the respective years (y- axis in the top graph). The change in proportion of different subtypes/CRFs over time was not significant for subtype G (p = 0.711, LBL), CRF43_02G (p = 0.497, LBL) and CRF02_AG (p = 0.323, LBL). However the increase of URFs over time was significant (p = 0.015, LBL). The y-axis on the bottom graph represents the proportions (%) of sequences collected over time for each subtype/CRF. The x-axis represents the time-period 1999-2013. The second y-axis on the bottom plot shows the HIV-1 prevalence in Nigeria over time.

Supplementary Figure S2. Potential recombination breakpoint hotspots detected in the pol alignment.

Small vertical lines at the bottom of the graph indicate breakpoint positions along the alignment. Breakpoints detected in each window of 300 nucleotides along the alignment were counted and plotted (solid line).

Supplementary Figure S3. Optimal cluster determined by the gap statistic method.

The gap statistic compares the total intra-cluster variation for different values of k with their expected values under null reference distribution of the data. This plot provides the gap statistic and standard error, identifying k=4 as the optimal cluster with the highest gap statistic.

Supplementary Figure S4. Groups of sequences with similar recombination breakpoint patterns that could be potential new CRFs.

Groups of sequences with similar recombination breakpoint patterns that could be potential new CRFs.

Supplementary Figure S5. Maximum likelihood phylogenetic tree of the pol region for Subtype G sequences (n = 203).

The blue braches indicate Nigerian sequences while the green indicate an SH-aLRT branch support above 0.9. The black branches indicate the non-Nigerian sequences. We observed one large NG cluster (in blue) that was considered for further analysis.

Supplementary Figure S6. Maximum likelihood phylogenetic tree for CRF02_AG sequences (n = 1170).

The blue braches indicate Nigerian sequences while the green indicate an SH-aLRT branch support above 0.9. The black branches indicate the non-Nigerian sequences. We observed 3 large Nigerian clusters (in blue) that were considered for further analysis.

Supplementary Figure S7. Maximum likelihood phylogenetic tree for CRF43_02G sequences (n = 667).

The blue braches indicate Nigerian sequences while the green indicate an SH-aLRT branch support above 0.9. The black branches indicate the non-Nigerian sequences. We observed one

large NG cluster (in blue) that was considered for further analysis. The purple sequences were later classified as URFs.

Supplementary Figure S8. Demographic history for the different Nigerian clusters. Pirate plots for the evolutionary rate in substitutions/site/year (3A) and time to the most recent common ancestor since 2013 (3B) for the five different colour-coded clusters in Nigeria. Each bean in the plot has a median estimate indicated as a bold horizontal black line and the Bayesian 95% highest posterior density interval as thin horizontal lines. The raw data from the sampling interval during Bayesian analysis is indicated by black dots inside the beans. The CRF43_02G had the rate parameter, root height and population sizes estimated with only the exponential growth model.

Supplementary Figure S1. Dynamics of subtype/CRF proportions, ART coverage and overall country HIV-1 prevalence over time.

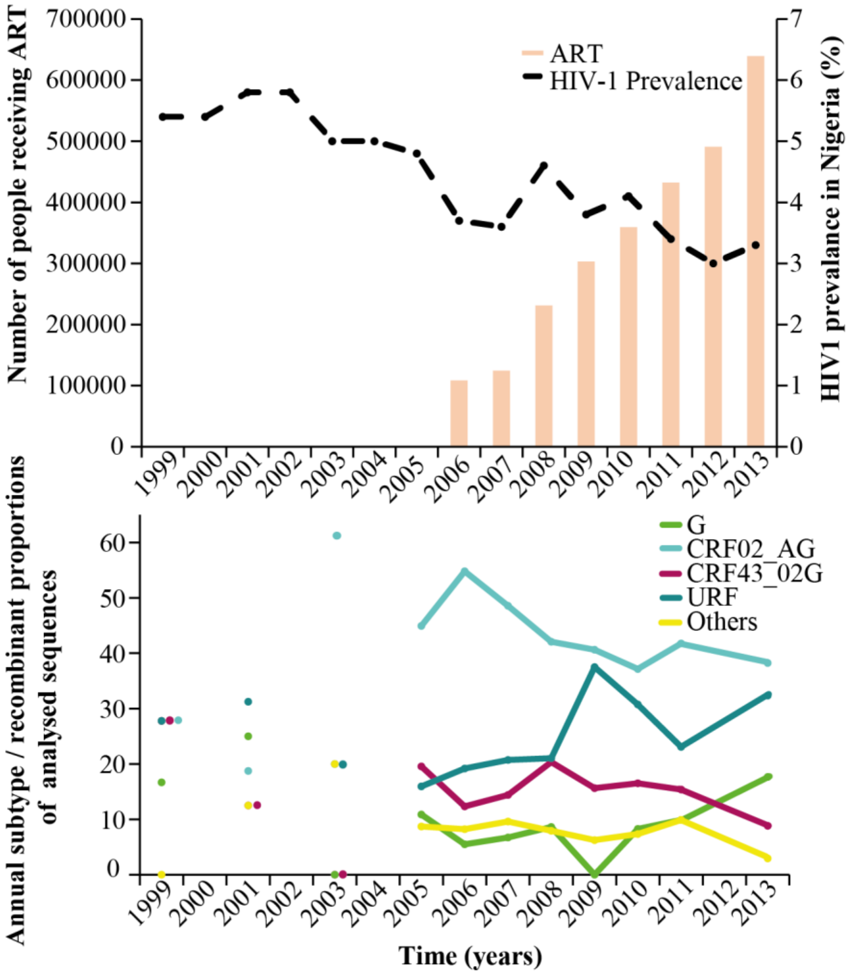

Supplementary Figure S2. Potential recombination breakpoint hotspots detected in the pol alignment.

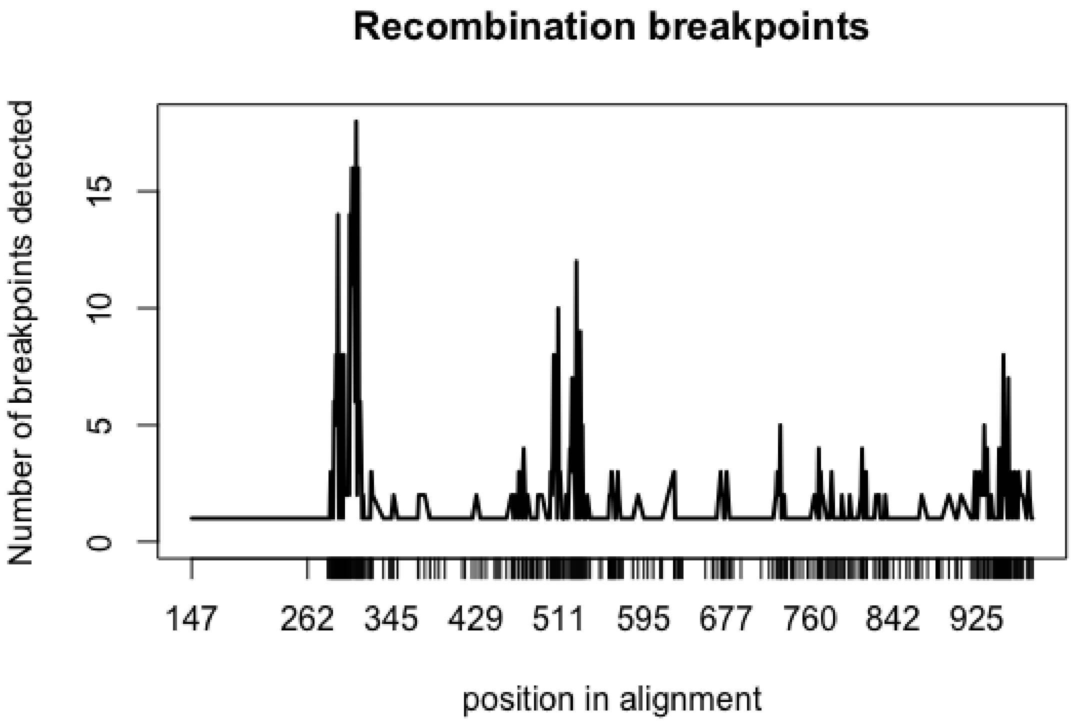

Supplementary Figure S3. Optimal cluster determined by the gap statistic method.

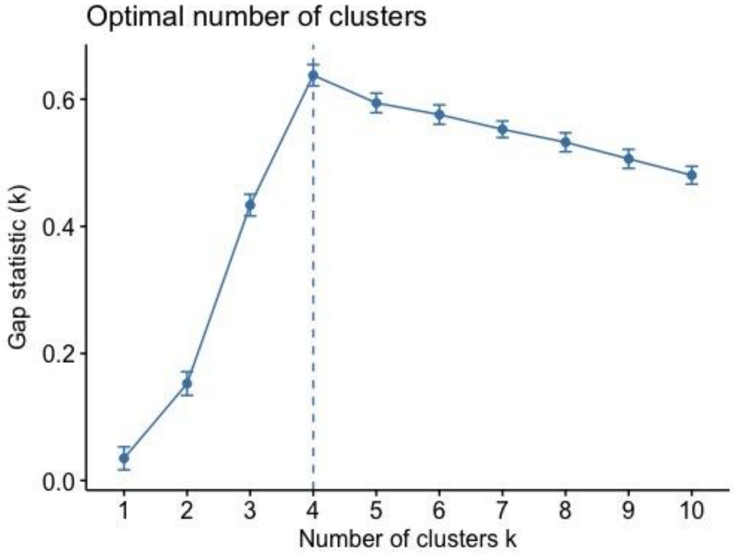

Supplementary Figure S4. Groups of sequences with similar recombination breakpoint patterns.

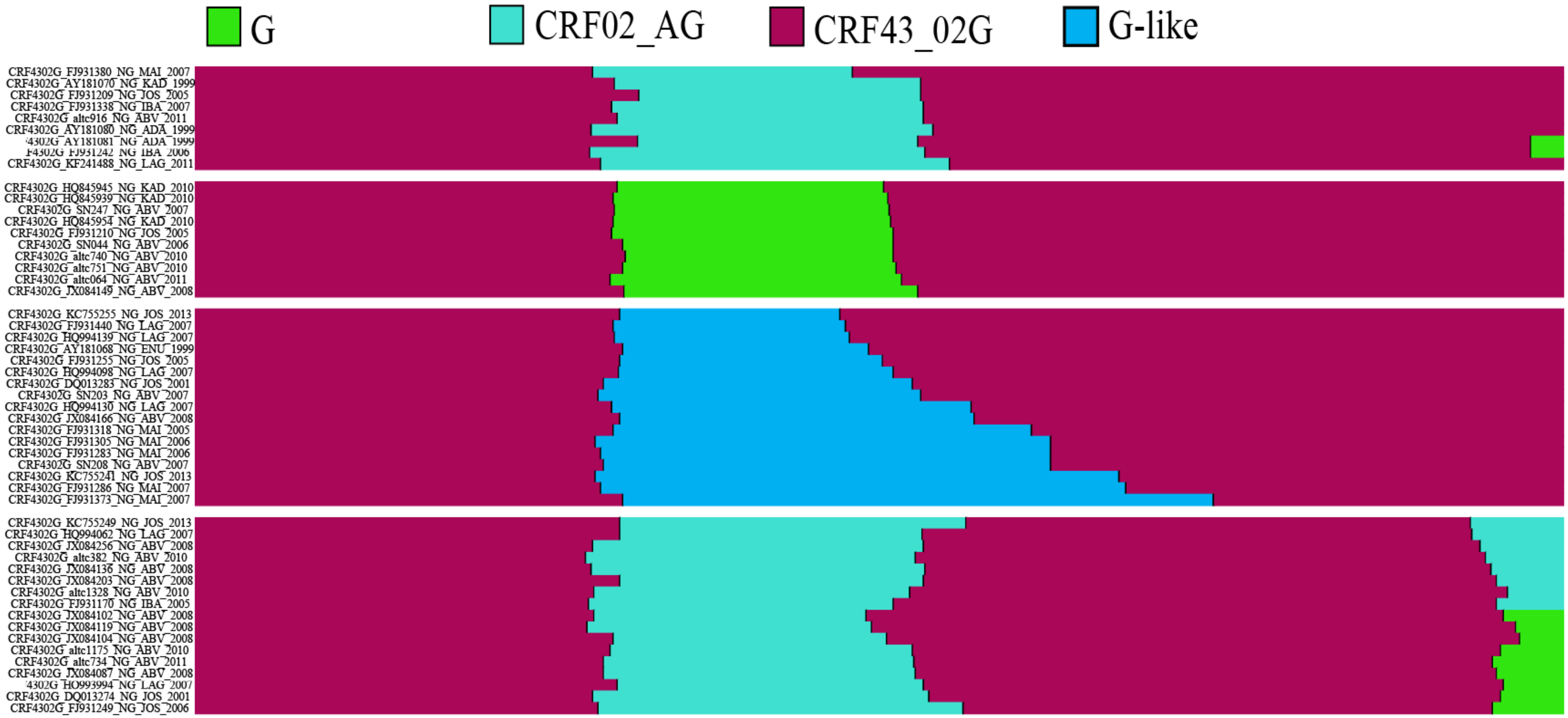

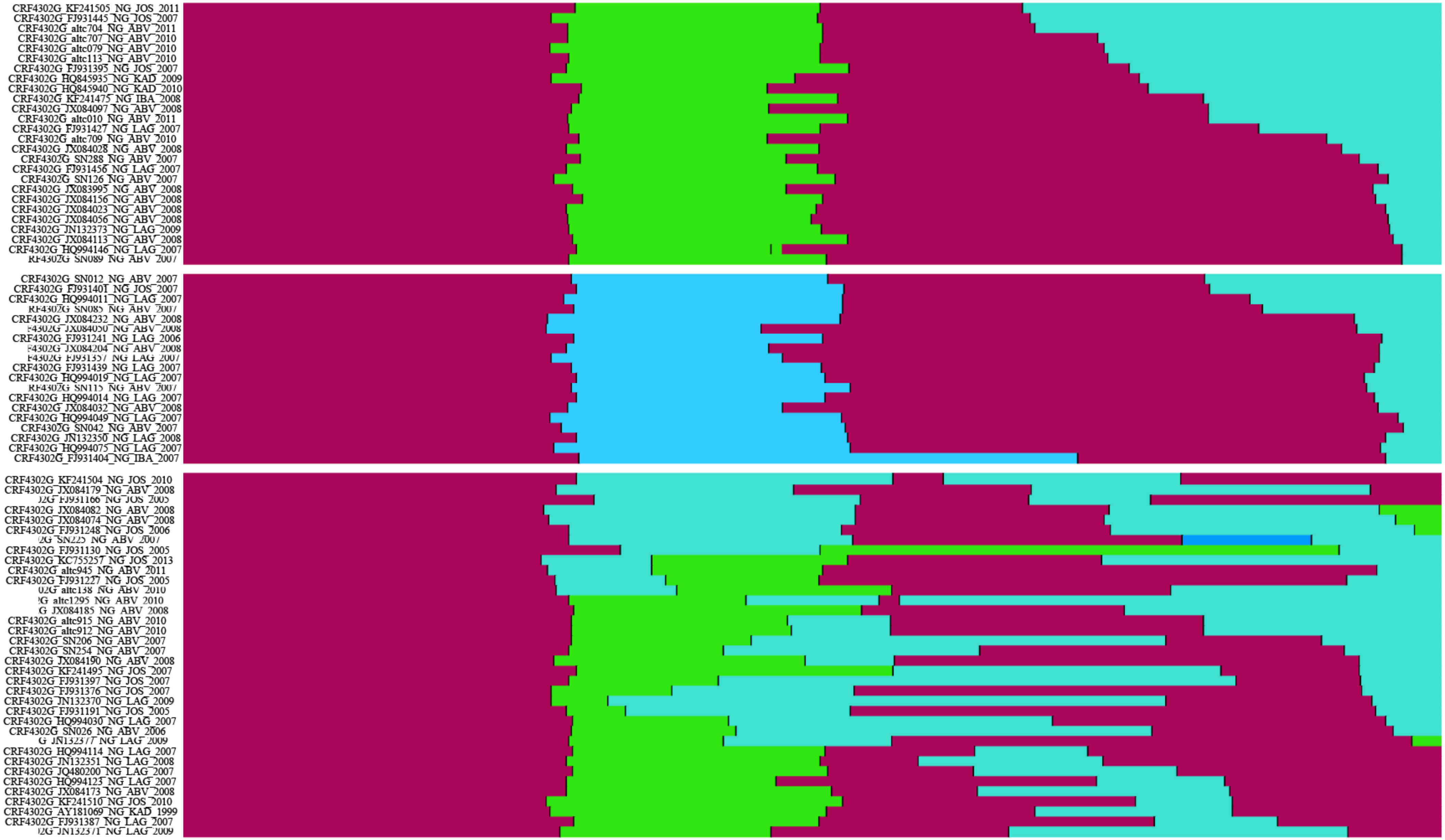
CRF4302G_KF241505_NG_JOS_2011
CRF4302G_FJ931445_NG_JOS_2007
CRF4302G_altc704_NG_ABV_2011
CRF4302G_altc707_NG_ABV_2010
CRF4302G_altc079_NG_ABV_2010
CRF4302G_altc113_NG_ABV_2010
CRF4302G_FJ931395_NG_JOS_2007
CRF4302G_HQ845935_NG_KAD_2009
CRF4302G_HQ845940_NG_KAD_2010
CRF4302G_KF241475_NG_IBA_2008
CRF4302G_JX084097_NG_ABV_2008
CRF4302G_altc010_NG_ABV_2011
CRF4302G_FJ931427_NG_LAG_2007
CRF4302G_altc709_NG_ABV_2010
CRF4302G_JX084028_NG_ABV_2008
CRF4302G_SN288_NG_ABV_2007
CRF4302G_FJ931456_NG_LAG_2007
CRF4302G_SN126_NG_ABV_2007
CRF4302G_JX083995_NG_ABV_2008
CRF4302G_JX084156_NG_ABV_2008
CRF4302G_JX084023_NG_ABV_2008
CRF4302G_JX084056_NG_ABV_2008
CRF4302G_JN132373_NG_LAG_2009
CRF4302G_JX084113_NG_ABV_2008
CRF4302G_HQ994146_NG_LAG_2007
RF4302G_SN089_NG_ABV_2007
CRF4302G_SN012_NG_ABV_2007
CRF4302G_FJ931401_NG_JOS_2007
CRF4302G_HQ994011_NG_LAG_2007
RF4302G_SN085_NG_ABV_2007
CRF4302G_JX084232_NG_ABV_2008
F4302G_JX084050_NG_ABV_2008
CRF4302G_FJ931241_NG_LAG_2006
F4302G_JX084204_NG_ABV_2008
F4302G_FJ931357_NG_LAG_2007
CRF4302G_FJ931439_NG_LAG_2007
CRF4302G_HQ994019_NG_LAG_2007
RF4302G_SN115_NG_ABV_2007
CRF4302G_HQ994014_NG_LAG_2007
CRF4302G_JX084032_NG_ABV_2008
CRF4302G_HQ994049_NG_LAG_2007
CRF4302G_SN042_NG_ABV_2007
CRF4302G_JN132350_NG_LAG_2008
CRF4302G_HQ994075_NG_LAG_2007
CRF4302G_FJ931404_NG_IBA_2007
CRF4302G_KF241504_NG_JOS_2010
CRF4302G_JX084179_NG_ABV_2008
2G_FJ931166_NG_JOS_2005
CRF4302G_JX084082_NG_ABV_2008
CRF4302G_JX084074_NG_ABV_2008
CRF4302G_FJ931248_NG_JOS_2006
2G_SN225_NG_ABV_2007
CRF4302G_FJ931130_NG_JOS_2005
CRF4302G_KC755257_NG_JOS_2013
CRF4302G_altc945_NG_ABV_2011
CRF4302G_FJ931227_NG_JOS_2005
02G_altc138_NG_ABV_2010
2G_altc1295_NG_ABV_2010
G_JX084185_NG_ABV_2008
CRF4302G_altc915_NG_ABV_2010
CRF4302G_altc912_NG_ABV_2010
CRF4302G_SN206_NG_ABV_2007
CRF4302G_SN254_NG_ABV_2007
CRF4302G_JX084190_NG_ABV_2008
CRF4302G_KF241495_NG_JOS_2007
CRF4302G_FJ931397_NG_JOS_2007
CRF4302G_FJ931376_NG_JOS_2007
CRF4302G_JN132370_NG_LAG_2009
CRF4302G_FJ931191_NG_JOS_2005
CRF4302G_HQ994030_NG_LAG_2007
CRF4302G_SN026_NG_ABV_2006
G_JN132377_NG_LAG_2009
CRF4302G_HQ994114_NG_LAG_2007
CRF4302G_JN132351_NG_LAG_2008
CRF4302G_JQ480200_NG_LAG_2007
CRF4302G_HQ994123_NG_LAG_2007
CRF4302G_JX084173_NG_ABV_2008
CRF4302G_KF241510_NG_JOS_2010
CRF4302G_AY181069_NG_KAD_1999
CRF4302G_FJ931387_NG_LAG_2007
2G_JN132371_NG_LAG_2009

Supplementary Figure S5. Maximum likelihood phylogenetic tree of the pol region for Subtype G sequences (n = 203).

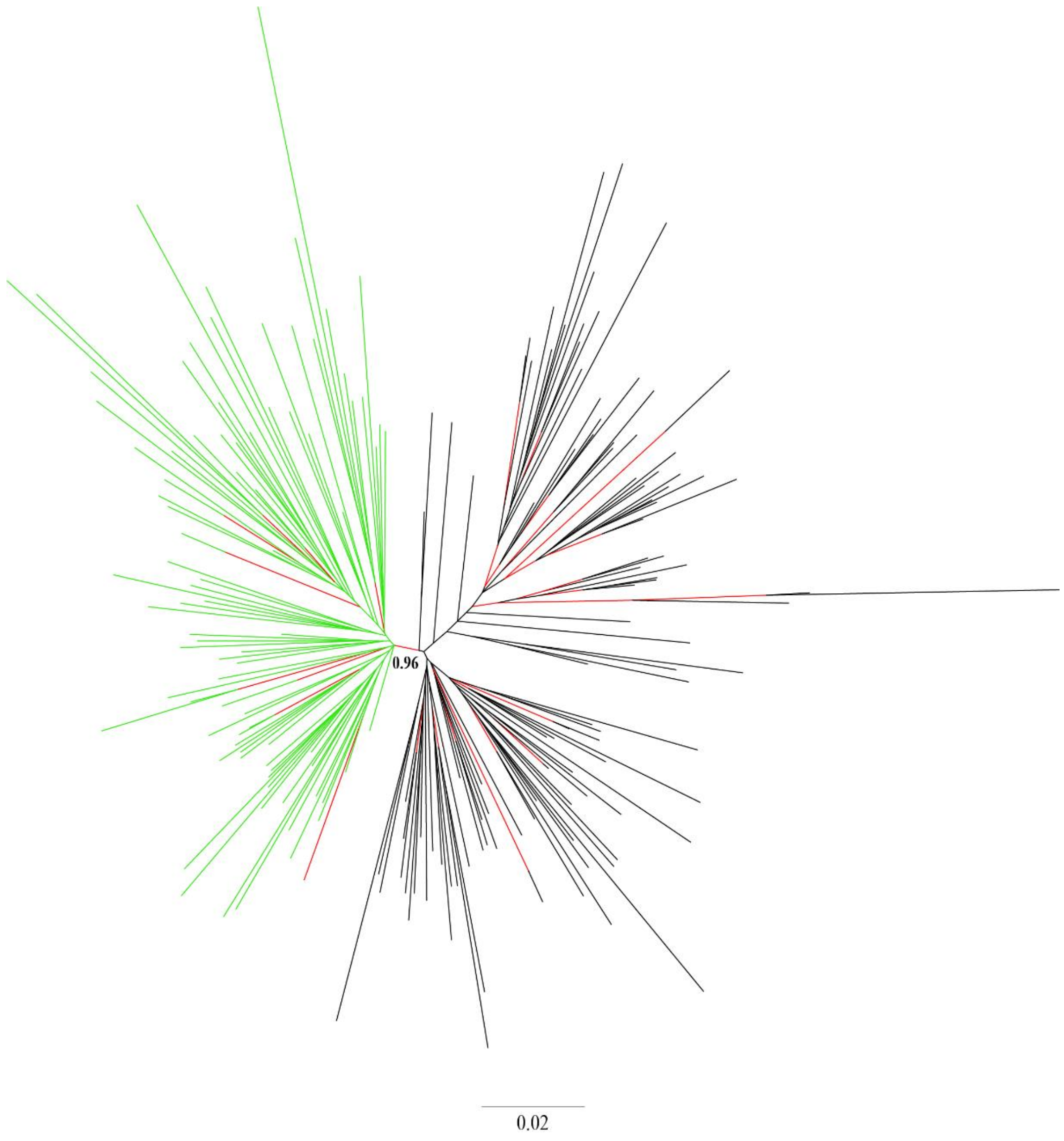

Supplementary Figure S6. Maximum likelihood phylogenetic tree for CRF02_AG sequences (n = 1170).

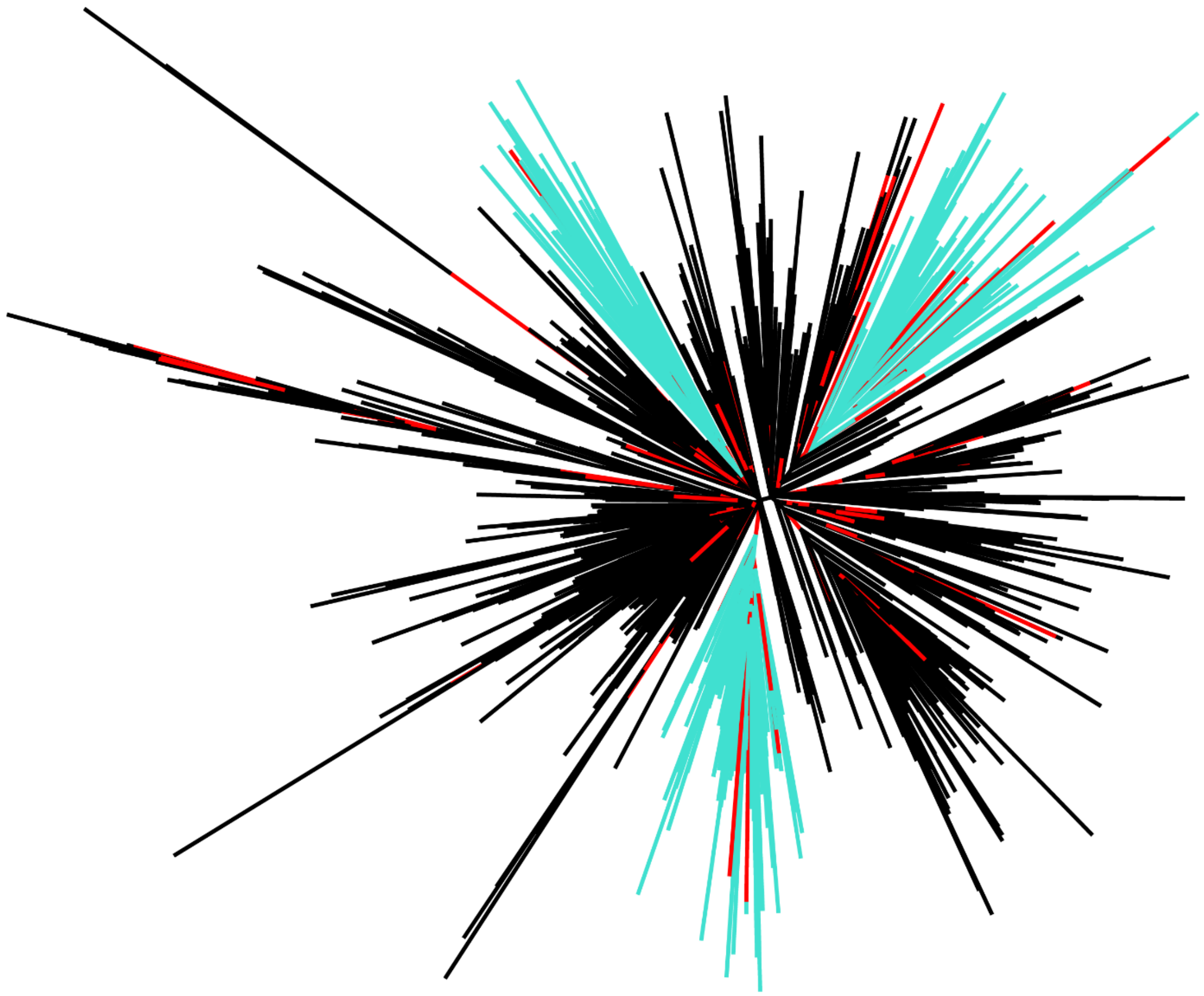

Supplementary Figure S7. Maximum likelihood phylogenetic tree for CRF43_02G sequences (n = 667).

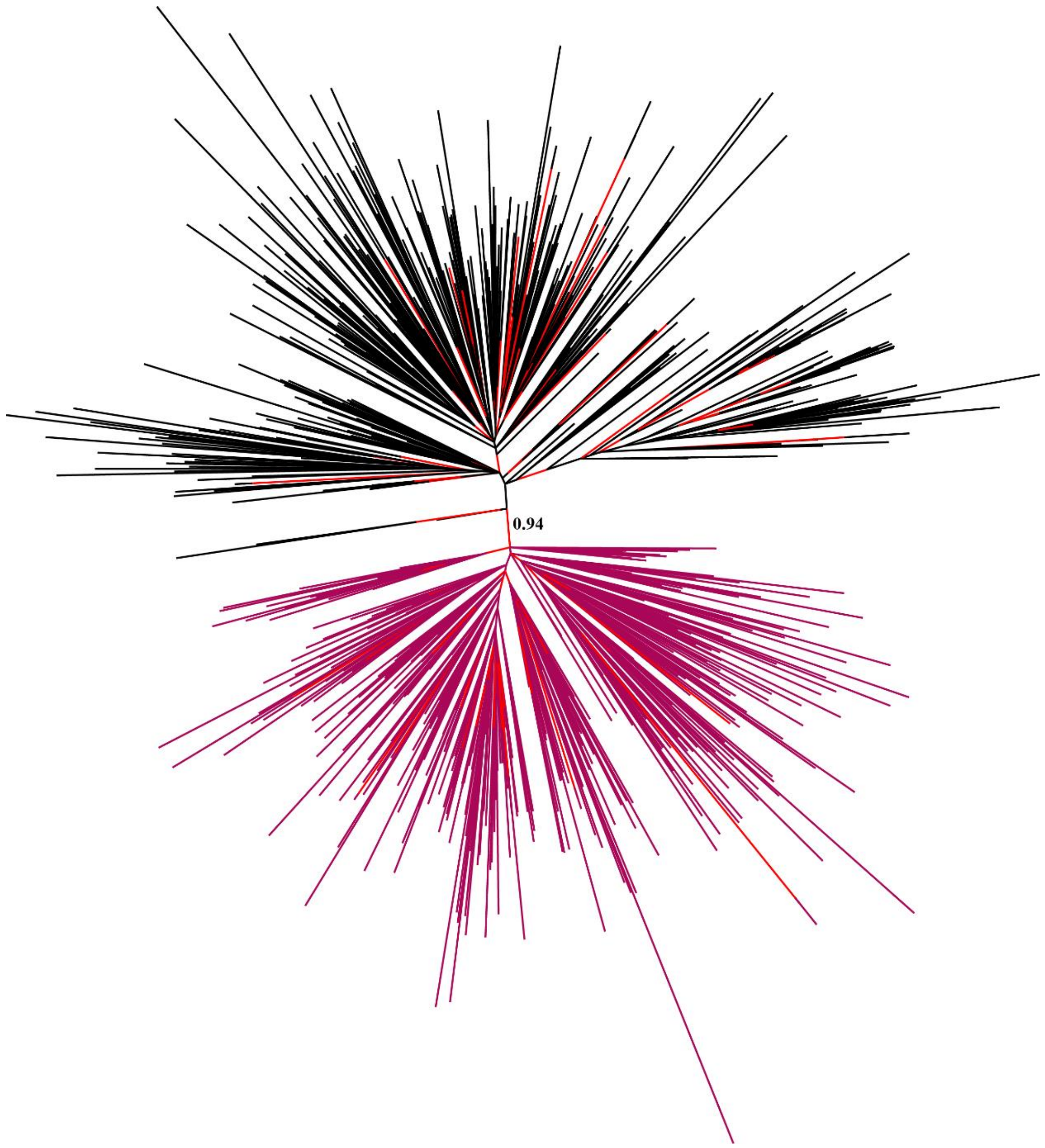

Supplementary Figure S8. Demographic history for the different Nigerian clusters.

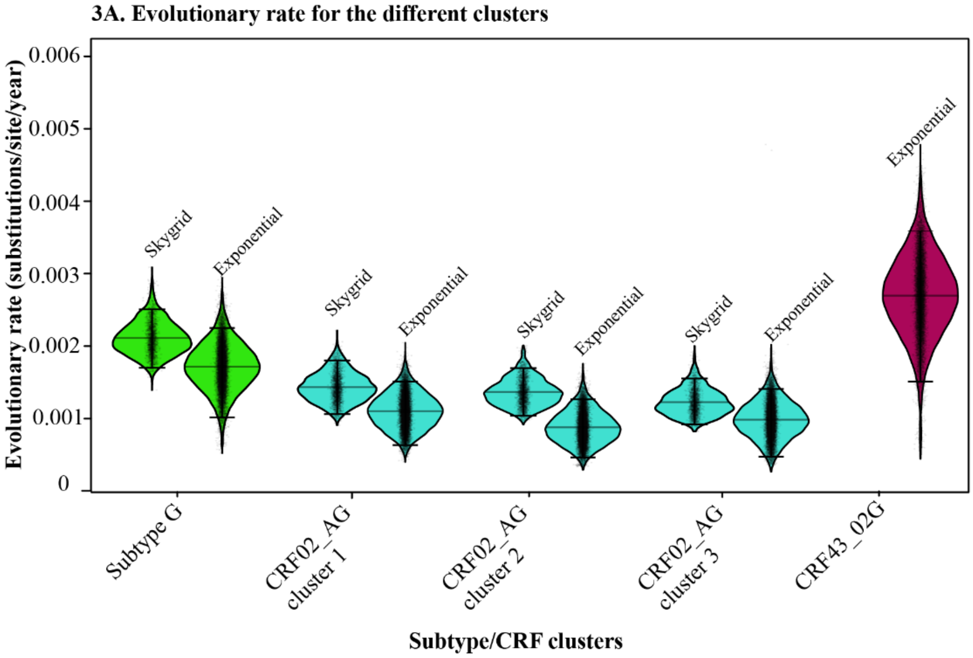

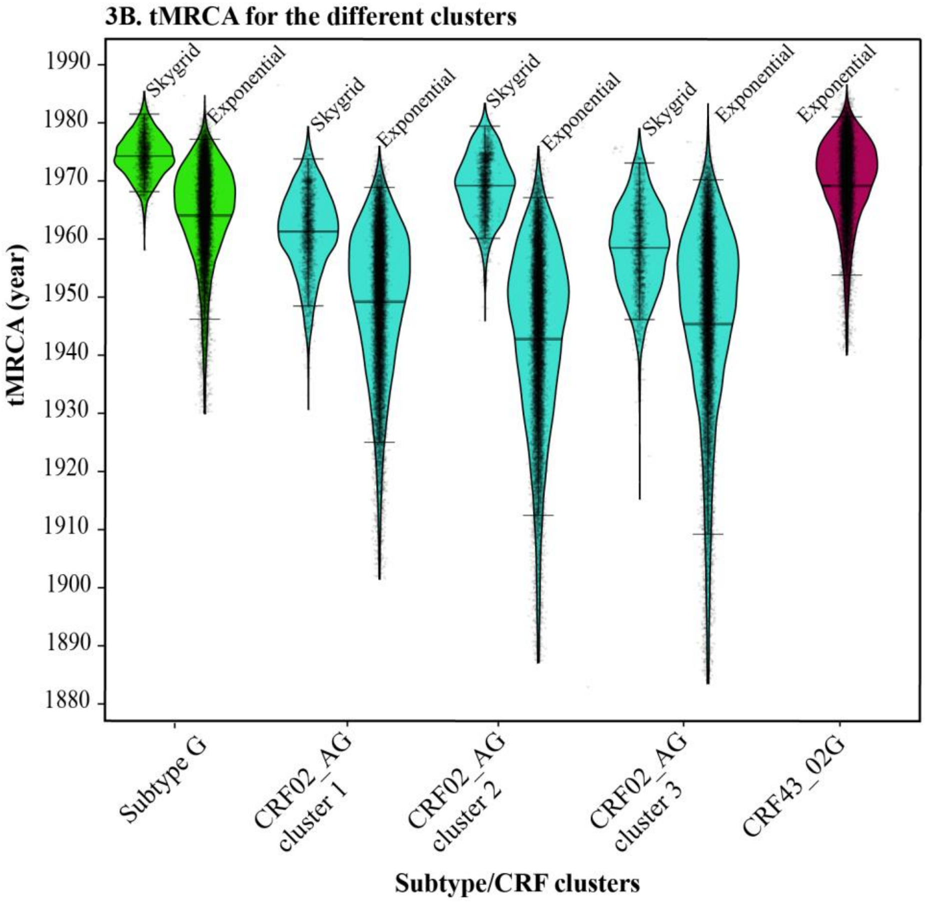

Supplement: Supplementary file 1 — Supplementary Information. [file 41598_2020_59944_MOESM1_ESM.pdf]
